# Are spousal carers’ perceptions of continuity and discontinuity within the relationship influenced by the symptoms of dementia?

**DOI:** 10.1177/1471301221994311

**Published:** 2021-02-08

**Authors:** Meryl A Lewis, Gerard A Riley

**Affiliations:** Centre for Applied Psychology, 1724 University of Birmingham, Birmingham, UK

**Keywords:** marriage, challenging interpersonal behaviour, communication, activities of daily living, relationships, cognition, depression, dementia

## Abstract

Some spousal carers experience their current relationship with the person with dementia as a continuation of the loving relationship they shared prior to the onset of dementia. For others, the experience is one of discontinuity; the prior relationship is lost and replaced with a different kind of relationship. The aim of this study was to investigate whether these differences are associated with particular symptoms of dementia. Thirty-five spousal carers completed the *Birmingham Relationship Continuity Measure*, the *Revised Memory and Behavior Checklist* (providing scores relating to cognitive decline, depression and challenging interpersonal behaviour), the *Communicative Effectiveness Index* and the *Bristol Activities of Daily Living Scale.* Experiencing discontinuity in the relationship was significantly correlated with communication difficulties, challenging interpersonal behaviour and the need for assistance in activities of daily living, but not with cognitive decline or depression. In a multiple regression, only the measures of challenging interpersonal behaviour and activities of daily living made significant unique contributions to the variance in continuity/discontinuity scores. Discontinuity is associated with reduced psychological well-being for the spousal carer and the provision of less person-centred care. Understanding which symptoms are more likely to lead to discontinuity allows the identification of those at risk of these experiences. Those at risk may require support to enable them to make sense of, and adjust to, certain symptoms of dementia in a way that has a less negative impact on their relationship.

## Introduction

Qualitative research involving partners/spouses who are providing care for someone with dementia has revealed considerable individual variation in how they experience their relationship. For some, the current relationship represents a continuation of the loving pre-dementia relationship, but others experience radical discontinuity – the pre-dementia relationship has been lost and replaced with something very different (Boylstein & Hayes, 2012; Chesla et al., 1994; Evans & Lee, 2014; Kaplan, 2001; Lindauer & Harvath, 2015; Quinn et al., 2015; Walters et al., 2010). For example, in her qualitative study, Kaplan (2001) described a number of couple types differentiated by the degree of continuity between the past and current relationship, ranging from the ‘till death do us parts’ (in which the sense of continuity is at its strongest) to ‘unmarried marrieds’ (in which the carer views themselves as being married in name only, and the pre-dementia relationship has been lost completely). It should be noted, of course, that not all pre-dementia relationships are loving, and this research focuses only on the experience of those who enjoyed a good relationship prior to the onset of dementia. It is also worth noting that an experience of continuity in the relationship does not preclude changes within the relationship and is not dependant on a denial of those changes. For some individuals, despite the changes and an acknowledgement of them, the essential core of a loving relationship persists, and the relationship is not experienced as being radically changed.

Based on a review of this qualitative research, Riley et al. (2013) described five distinct but closely related dimensions of these two contrasting experiences of continuity and discontinuity: *same/different feelings, same/different person*, *couplehood, relationship redefined* and *loss*. *Same/different feelings* captures the notion that, in continuity, the carer still feels the same love and affection for their partner as before, whereas in discontinuity, these feelings have diminished and been replaced by other feelings such as protectiveness, emotional distancing or even dislike. A major contributor to changes in feeling towards the other person in discontinuity appears to be a sense that the person with dementia is no longer the same person and has become a stranger. In continuity, by contrast, the person with dementia is experienced as being essentially the same, despite changes that have inevitably occurred (*same/different person*). The dimension of *couplehood* describes a difference between continuity and discontinuity in terms of whether the carer feels part of a couple. In discontinuity, the sense of living, enjoying and coping with life together, as a couple, is lost and replaced by a more individualistic perspective, whereas in continuity, the sense of belonging to a couple is retained. In continuity, the carer also experiences the relationship as a continuation of the marriage/partnership they enjoyed before the onset of dementia, but in discontinuity, the relationship feels radically different and is experienced as a relationship defined by the giving and receiving of care, more akin to that between a nurse and patient (*relationship redefined*). Finally, in discontinuity, the experience of losing the person and relationship as they were before the onset of dementia is associated with a sense of loss and grief, an experience that is more limited or absent in the case of continuity (*loss*). Riley et al. (2013) described the development and psychometric evaluation of a questionnaire to measure these five dimensions of relationship continuity/discontinuity (the *Birmingham Relationship Continuity Measure*).

Compared to discontinuity, continuity appears to have some benefits (Riley, 2019). Qualitative studies have suggested it may be linked with better emotional well-being for the caregiving partner (Boylstein & Hayes, 2012; Walters et al., 2010). More recent quantitative studies using the *Birmingham Relationship Continuity Measure* have supported this. Scores indicating greater continuity are associated with lower levels of anxiety and depression, less sense of burden and the derivation of greater satisfaction from the caregiving role (Poveda et al., 2017; Riley et al., 2018). Qualitative and mixed-method studies have also suggested that continuity is associated with a more person-centred approach to the understanding and management of challenging interpersonal behaviours (Chesla et al., 1994; Lewis, 1998; Murray & Livingston, 1998; Riley et al., 2020; Walters et al., 2010).

Given the potential benefits of continuity, it is important to understand why some partners experience continuity, but others experience discontinuity. Such knowledge would permit the identification of those at risk of discontinuity and enable them to be supported at an earlier stage in preserving the core of their relationship. Knowing why these different experiences arise would also deepen understanding of their nature and thereby enable the development of more effective support.

One reason for differences in continuity/discontinuity may relate to the symptoms of dementia shown by the person with dementia. It may be more difficult to maintain a sense of continuity with the pre-dementia relationship in the face of certain changes and losses than others. In qualitative research on acquired brain injury, it has been suggested that discontinuity may be particularly likely when the person with the brain injury shows challenging interpersonal behaviours such as aggression and attempts to control the other person, and when they lack emotional warmth and responsiveness (Bodley-Scott & Riley, 2015; Villa & Riley, 2017). A lack of emotional warmth and responsiveness refers to the relative infrequency of communications and behaviours that indicate the presence of positive feelings towards the carer, and encompasses both spontaneous communications/behaviours and those in response to what the carer does and says. The link between this apparent emotional indifference and perceptions of changed identity has also been highlighted in a qualitative study involving participants living with dementia (Boylstein & Hayes, 2012).

Two quantitative studies also provide some support for the suggestion that discontinuity is more likely to occur in the presence of challenging interpersonal behaviour and lack of emotional warmth compared to other symptoms, although the evidence is somewhat mixed. Spousal carers in a study by Poveda et al. (2017) completed the *Neuropsychiatric Inventory* (Cummings et al., 1994) which evaluates symptoms of psychosis and changes in mood and behaviour. Discontinuity (measured by the *Birmingham Relationship Continuity Measure*) was significantly correlated with the total score on the inventory. It was uncorrelated with items related to mood and symptoms of psychosis, but significantly correlated with items related to apathy (which may be viewed as an aspect of emotional unresponsiveness) and to disinhibition and agitation (which may be viewed as challenging interpersonal behaviours). However, it was uncorrelated with irritability, which would also be considered challenging interpersonal behaviour. Participants in the study also completed *The Awareness of Social Inference Test* (McDonald et al., 2003) which was used to measure aspects of the social cognition of the person with dementia (specifically, their ability to read facial expressions and their ability to use paralinguistic cues, such as tone of voice, in order to interpret conversational meaning). An ability to read the emotional state of others presumably has an impact on how emotionally responsive the person with dementia is to their partner. However, scores on the *Birmingham Relationship Continuity Measure* were uncorrelated with scores on this test.

In another quantitative study, Strohminger and Nichols (2015) required family members of people with Alzheimer’s or fronto-temporal dementia to rate the extent to which the person with dementia was the same person, alongside questionnaires about the presence of common symptoms of dementia and changes in personality. In analyses using structural equation modelling, the presence of symptoms indicating a loss of ‘morality’ (defined as the ability to judge right from wrong and the capacity to be moved by the suffering of others) and changes in ‘moral’ personality traits (e.g. loss of empathy) were the only factors that significantly predicted identity change in both groups. Changes in cognition, mood, motivation and other challenging interpersonal behaviours (e.g. paranoia) were not significant predictors. The results thus suggested that loss of ‘morality’ (which could be viewed as an aspect of challenging interpersonal behaviours) and empathy (which could be viewed as an aspect of emotional warmth) may be particularly associated with perceptions of changed identity.

These two previous quantitative studies are limited in some respects. The study by Poveda et al. (2017) examined a relatively narrow range of symptoms, and the study by Strohminger and Nichols (2015) focused only on discontinuity of the identity of the person with dementia rather than the wider concept of relationship discontinuity. The present study expanded these investigations by assessing the association between scores on the *Birmingham Relationship Continuity Measure* and a wider range of measures of dementia symptoms. Alongside measures of communication, mood and challenging interpersonal behaviours, partners also completed questionnaires about their partner’s cognitive decline and loss of ability to complete activities of daily living. The expectation was that, relative to other symptoms, discontinuity would be particularly associated with challenging interpersonal behaviours and a lack of emotional responsiveness.

## Method

Ethical approval for the study was provided by the STEM Ethical Review Committee of the University of Birmingham (reference number ERN-14-1398). All participants provided written informed consent.

### Recruitment and participants

Oral presentations about the research were given to groups of carers in a range of organisations providing services to people with dementia and their families. Those interested were invited to speak individually with the researcher following the presentation. They were given an opportunity to ask questions and provided with more detailed written information to take away. Using contact details they provided, the researcher subsequently got in touch with them to ask if they wanted to participate.

The presentations and written information provided details about the inclusion and exclusion criteria. Participants were required to be currently living with, and caring for, a spouse/partner with a diagnosis of dementia; to have been in the relationship at least 5 years before the diagnosis of the dementia; and to be capable of giving informed consent and completing questionnaires written in English. People were excluded if the diagnosis of dementia had been given less than 4 months before participation and if, prior to the dementia starting, they were already providing care to their partner because of a learning disability, a serious medical condition or mental health difficulties.

A power analysis was carried out to establish a minimum requirement for the sample size. The primary statistical analysis involved correlations. According to G*Power (Faul et al., 2007), with alpha set at .05 and power at .8 in a two-tailed test, a sample size of at least 29 would be required to detect a large correlation (r = .5).

### Measures and procedure

Relationship continuity was assessed using the *Birmingham Relationship Continuity Measure* which was reported in the initial evaluation study to have good internal consistency, test–retest reliability and construct validity (Riley et al., 2013). Higher scores indicate more continuity. Measures of dementia symptoms were selected to cover a broad range. The *Revised Memory and Behavior Checklist* (Teri et al., 1992) provides an evaluation of the frequency of, and distress caused by, problems with memory/concentration, depression and challenging interpersonal behaviours (labelled ‘disruption’). Separate scores are calculated for each of these three areas of difficulty, and higher scores indicate a greater frequency of the difficulty. The measure of distress caused by the symptoms was not included in the present study: The focus of the study was on investigating the association between continuity/discontinuity and a range of symptoms, not on the distress caused by those symptoms. Furthermore, the other symptom measures included in the study do not assess the distress caused by the symptoms and so comparison of distress across the range of symptom measures would not have been possible. The *Bristol Activities of Daily Living Scale* (Bucks et al., 1996) provides an evaluation of the functional abilities of the person with dementia in relation to activities such as self-care, household tasks and recreation. Higher scores indicate greater dependency. It was not possible to identify any carer-rated measures assessing emotional warmth that has been validated in dementia research. As a substitute, the *Communicative Effectiveness Index* (Lomas et al., 1989) was used. This assesses loss of ability in everyday communication behaviours and includes an item about communicating emotions, alongside other items such as taking part in a conversation. It was originally designed for use with people experiencing communication difficulties after a stroke and the wording was changed where necessary to reflect its use in dementia. Higher scores indicate greater impairment.

Along with the symptom measures, participants also completed the *Relationship Assessment Scale* (Hendrick, 1988) with reference to their pre-dementia relationship (i.e. before the onset of any symptoms). As explained in the Introduction, the concept of relationship continuity refers to the continuance of a loving pre-dementia relationship. Clearly, not all pre-dementia relationships are loving, and this presents a potential source of confounding in the results. To address this, the *Relationship Assessment Scale* was used to provide a measure of the quality of the pre-morbid relationship. If there were any participants who reported an unsatisfactory pre-dementia relationship, the intention was to run the analysis without them. The measure has seven items rated on a five-point Likert scale with three points anchored to verbal descriptions, ranging from 1 (designated ‘poor’) through 3 (‘average’) to 5 (‘excellent’), with higher scores indicating better quality. A score of 21 represents an ‘average’ score for the seven items and so a score below 21 was set as the exclusion criterion.

Participants were given the option of completing the questionnaires at home or at the premises of the organisation from which they were recruited, and the option of completing them with a researcher present or completing them alone. These options were given to enable the participation of those who might otherwise be discouraged or prevented from taking part. In the case of those who chose to complete the questionnaires with a researcher present, the researcher was available to clarify any questions the participant had about the questionnaires and to read the items aloud for those with visual impairment.

## Results

Thirty-six full data sets were returned, but one of these was excluded because one of the inclusion criteria was not met (specifically, the person had not lived with the person with dementia for at least 5 years prior to the onset of dementia). The final sample thus comprised 35 participants. Demographic- and dementia-related information is summarised in Table 1. Table 2 presents the descriptive statistics for each of the questionnaires. Except for *Revised Memory and Behavior Checklist*-*Memory*, all the questionnaires showed an acceptable level of internal consistency (i.e. Cronbach’s alpha was at least .7). On the *Relationship Assessment Scale*, all participants scored above the cut-off point of 21, indicating that all were generally satisfied with their pre-dementia relationship. Exclusion of participants from the analysis on the basis of an unsatisfactory pre-dementia relationship was, therefore, not required.

**Table 1.** Demographic information (*N* = 35).

Participant characteristics
Gender: 26 female; 9 male
Ethnicity: 32 white British; 3 others
Age: Mean = 73; standard deviation = 8; range = 54–87
Employment status: 4 still in some form of employment
Characteristics of person with dementia
Gender: 9 female; 26 male
Ethnicity: 33 white British; 2 others
Age: Mean = 76; standard deviation = 8; range = 56–91
Employment status: 0 in employment
Characteristics of relationship
Orientation: 35 heterosexual
Length of relationship (years): Mean = 48; standard deviation = 15; range = 7–75^a^
Characteristics of dementia
Type: Vascular = 13; Alzheimer’s = 7; mixed = 6; unknown/other = 9
Time since diagnosis (months): Mean = 42; standard deviation = 29; range = 5–120

^a^Some participants provided information about how long they had known the person with dementia, rather than how long they had been married/partners.

**Table 2.** Descriptive statistics.

Scale	Number of items	Possible range	Observed range	Mean	Standard deviation	Cronbach’s alpha
BRCM	23	23–115	23–114	58.43	26.89	.96
CEI	16	16–80	21–77	58.23	14.71	.93
RMBC-memory	7	0–7	2–7	5.80	1.30	.55
RMBC-depression	9	0–9	0–8	3.83	2.68	.86
RMBC-disruption	8	0–8	0–8	2.51	2.15	.77
BADLS	20	0–60	4–58	27.99	10.96	.91
RAS	7	7–35	24–35	31.94	3.30	.72

BRCM: Birmingham Relationship Continuity Measure; CEI: Communicative Effectiveness Index; RMBC: Revised Memory and Behaviour Checklist; BADLS: Bristol Activities of Daily Living Scale; RAS: Relationship Assessment Scale (for pre-dementia relationship).

Distributions were checked for univariate outliers and departures from normality. One outlier was detected on the *Bristol Activities of Daily Living Scale*, but no adjustments were made because inclusion of the score did not have any noticeable impact on the outcome of the analysis. Distributions on the *Revised Memory and Behavior Checklist-Memory* and *Disruption* scores departed significantly from normal. Logarithmic transformations were used to correct this, although this was not entirely successful for the *Memory* score, and the results for this variable should be interpreted with some caution. There were no multivariate outliers. The data otherwise met the assumptions for parametric analysis, and Pearson’s correlation coefficient was calculated.

Correlations are shown in Table 3. As expected, *Birmingham Relationship Continuity Measure* scores were significantly associated with the *Communicative Effectiveness Index* and the frequency of ‘disruptive behaviour’ measured by the *Revised Memory and Behavior Checklist*, but not with memory or mood changes. Unexpectedly, the *Birmingham Relationship Continuity Measure* scores also correlated significantly with the *Bristol Activities of Daily Living Scale*.

**Table 3.** Correlations.

	BRCM	CEI	RMBC-memory	RMBC-depression	RMBC-disruption
CEI	−.503*				
	*p* = .002				
RMBC-memory	−.218	−.089			
	*p* = .208	*p* = .612			
RMBC-depression	.106	−.091	.175		
	*p* = .544	*p* = .602	*p* = .315		
RMBC-disruption	−.586*	.177	.098	.174	
	*p* < .001	*p* = .310	*p* = .574	*p* = .318	
BADLS	−659*	.684*	−.131	−.187	.261
	*p* < .001	*p* < .001	*p* = .452	*p* = .281	*p* = .130

*p < .05. BRCM: Birmingham Relationship Continuity Measure; CEI: Communicative Effectiveness Index; RMBC: Revised Memory and Behaviour Checklist; BADLS: Bristol Activities of Daily Living Scale.

Correlations amongst the symptom variables were generally small. However, there was a large significant correlation between the *Bristol Activities of Daily Living Scale* and the *Communicative Effectiveness Index* (Table 3). This raised the possibility that the association between one of these variables and the *Birmingham Relationship Continuity Measure* may have occurred simply because of its association with the other symptom variable. To investigate this further, the three symptom variables that were significantly correlated with the *Birmingham Relationship Continuity Measure* (i.e. the *Revised Memory and Behavior Checklist-Disruption*, *Bristol Activities of Daily Living Scale* and *Communicative Effectiveness Index*) were entered as predictors in a multiple regression, with the *Birmingham Relationship Continuity Measure* as the outcome variable. The analysis was restricted to these three predictors because a power analysis using G*Power indicated that the sample size of the study was inadequate to test the contribution of more than three (with power set at .80 and alpha at .05, a sample of 36 is required to detect large effects (f^2^ = .35) of three predictors). The results are shown in Table 4. Both the *Revised Memory and Behavior Checklist*-*Disruption* and *Bristol Activities of Daily Living Scale* variables made a significant unique contribution to the variance in *Birmingham Relationship Continuity Measure* scores, but the *Communicative Effectiveness Index* did not. Overall, the predictors explained 59% of the variance (adjusted R^2^ = .588).

**Table 4.** Regression analysis, BRCM as the outcome variable.

	Standardised coefficient beta	*p*-value	Part correlation
CEI	−.099	.515	−.072
RMBC-distribution	−.445	<.001*	−.430
BADLS	−.475	.004*	−.340

*p < .05. BRCM: Birmingham Relationship Continuity Measure; CEI: Communicative Effectiveness Index; RMBC: Revised Memory and Behaviour Checklist; BADLS: Bristol Activities of Daily Living Scale.

Associations between the *Birmingham Relationship Continuity Measure* and each of the demographic, relationship and dementia-related variables were evaluated using correlations for continuous variables and one-way ANOVA for categorical variables. Due to insufficient numbers in each group, no analyses were performed for ethnicity. *Birmingham Relationship Continuity Measure* scores were not related significantly to any of the other variables, including gender, type of dementia, time since diagnosis, duration of relationship and quality of the pre-dementia relationship (measured by the *Relationship Assessment Scale*).

## Discussion

As expected, scores on the continuity measure showed a significant negative correlation with communication difficulties and challenging interpersonal behaviour, but not with cognitive decline or depression. Unexpectedly, there was also a significant negative correlation with the need for assistance in activities of daily living. In a multiple regression, only the measures of challenging interpersonal behaviour and activities of daily living made significant unique contributions to the variance in scores on the continuity measure.

### Challenging interpersonal behaviours

The association between discontinuity and challenging interpersonal behaviour is consistent with qualitative research in acquired brain injury linking discontinuity with challenging interpersonal behaviour such as aggression (Bodley-Scott & Riley, 2015; Villa & Riley, 2017) and with the dementia study of Poveda et al. (2017) in which the *Birmingham Relationship Continuity Measure* was significantly negatively correlated with the *Neuropsychiatric Inventory*, which has some overlap with *Revised Memory and Behavior Checklist*-*Disruption* in terms of items relating to aggression, agitation and disinhibition. A more recent study also reported a large significant negative correlation between *Birmingham Relationship Continuity Measure* scores and the *Behaviour* subscale of the *Caregiver Hassles Scale* which assesses challenging interpersonal behaviours (Riley et al., 2020). The result is also consistent with the study of Strohminger and Nichols (2015) in which losses in ‘morality’ and ‘moral personality’, but not changes in cognition, mood, motivation or behaviours such as agitation, were associated with perceptions of identity change in the person with dementia.

The link between challenging interpersonal behaviours and experiences of discontinuity requires further exploration. A qualitative approach may be preferable for exploring the nature of such a complex connection. In the context of a loving pre-dementia relationship, challenging interpersonal behaviours such as aggression presumably seem very inconsistent with the person and the relationship as they were before the onset of dementia. Perhaps this inconsistency makes it particularly difficult to maintain a sense of continuity. Another reason for the link may be that such behaviours often elicit strong negative emotional responses in the carer towards the person with dementia, such as feeling hurt, angry and fearful. Participants in the study by Bodley-Scott and Riley (2015) described how it was difficult to switch off such feelings in favour of more positive feelings such as love and intimacy. Consistent with this, several quantitative studies in dementia have reported that higher levels of challenging interpersonal behaviour are associated with reduced levels of warmth and intimacy on the part of the carer within the relationship (De Vugt et al., 2003; Spector et al., 2016; Spruytte et al., 2002). A loss of love and affection may, in turn, make it difficult to retain a sense of continuity with the pre-dementia relationship.

### Cognition and mood

The depression and memory components of the *Revised Memory and Behavior Checklist* were not correlated with the *Birmingham Relationship Continuity Measure*. Most carers are likely to have experienced some degree of low mood in the person with dementia during the period before the onset of dementia. Low mood may also be seen by the carer as an understandable reaction to having dementia (Aminzadeh et al., 2007). It may be, then, that low mood is not viewed as alien to the essential character of the person with dementia in the way that changes such as aggression are. As such, it may have less of an impact on the carer’s perception of the identity of the person with dementia. Cognitive losses may present a greater contrast with the person before the onset of dementia, but they are perhaps less central to the identity of that person. Strohminger and Nichols (2014) presented members of the public with hypothetical scenarios in which some event caused a range of personal changes to a fictional character. Participants were required to say whether the character remained the same person. Changes to basic perceptual and cognitive abilities were significantly less likely to result in judgements that the character was no longer the same person than changes to moral and other personality traits.

### Communication

Although *Birmingham Relationship Continuity Measure* scores were significantly associated with the *Communicative Effectiveness Index*, this association disappeared when the correlation of the questionnaire with the *Bristol Activities of Daily Living Scale* was taken into account in the multiple regression. This uncertainty is reflected in other literature about this issue. Although emotional warmth and responsiveness are highlighted in the qualitative brain injury literature as contributing to discontinuity (Bodley-Scott & Riley, 2015; Villa & Riley, 2017), Poveda et al. (2017) found no correlation between the *Birmingham Relationship Continuity Measure* and a measure of social cognition. In the study by Strohminger and Nichols (2015), aphasia was not a significant predictor of ratings of identity change, but empathy (which overlaps to some extent with the notion of emotional warmth and responsiveness) was included in the construct of ‘moral’ symptoms that was the only significant predictor. Perhaps, the resolution of this uncertainty lies in considering the measures used. Although *The Awareness of Social Inference Test* used by Poveda et al., the aphasia item used by Strohminger and Nichols and the *Communicative Effectiveness Index* used in the present study are measuring skills that can contribute to the expression of emotional warmth and responsiveness, none offers a direct and comprehensive measure of this ability. The links between lack of emotional warmth and discontinuity merit further investigation using a measure with greater validity. However, as noted earlier, we were unable to identify a carer-rated measure assessing emotional warmth that has been validated in dementia research.

### Activities of daily living

An unexpected finding was that discontinuity was associated with greater difficulties in carrying out activities of daily living, and that this association was significant even when its correlation with the *Communicative Effectiveness Index* was taken into account in the multiple regression. This is consistent with a recent study (Riley et al., 2020) in which *Birmingham Relationship Continuity Measure* scores showed a significant moderate correlation with a different measure of difficulties with activities of daily living (the *Caregiver Hassles Scale*). The reason for this association is unclear. Due to the range and complexity of abilities required in carrying out everyday activities, the *Bristol Activities of Daily Living Scale* may provide a reasonable index of *global* impairment (i.e. the number and magnitude of differences between the person as they were before the dementia and as they are now). Previous qualitative research presents a seemingly inconsistent picture of the association between discontinuity and the severity of global impairment. Although some studies have suggested that continuity becomes increasingly difficult to maintain as the level of global disability increases (Gillies, 2012), others have reported the maintenance of continuity even when the person with dementia is very severely disabled and the occurrence of discontinuity even when the level of disability is relatively mild (Chesla et al., 1994; Kaplan, 2001). The explanation of this inconsistency may be that global impairment makes continuity more difficult, but other factors (such as challenging interpersonal behaviours) are also involved. Consequently, although there may be a tendency for discontinuity to increase as global impairment increases, there will still be considerable individual variation within this, and some people may experience discontinuity even when global disability is relatively mild, while others are able to maintain continuity even when it is relatively severe. There is some support for this in the present study: The person who reported the highest score on the *Bristol Activities of Daily Living Scale* (58 out of 60, indicating complete dependency on all but one item) nevertheless scored at the median on the *Birmingham Relationship Continuity Measure* (i.e. only half of the sample showed more continuity). Conversely, compared to the person with the lowest score on the *Birmingham Relationship Continuity Measure*, 66% of the sample was looking after people who needed more support in activities of daily living.

### Limitations

Some limitations of the study should be noted. The sample was non-random, self-selected and homogenous in terms of demographic characteristics. Care should, therefore, be taken in generalising the findings. Sample size was also relatively small and more modest relationships amongst the variables may not have been detected because of this. This includes correlations between continuity/discontinuity and demographic/other factors, such as the type of dementia. The correlational design precludes any firm conclusions about causality: Possible alternative explanations of the association between certain symptoms and continuity/discontinuity are that those who perceive their relationship as discontinuous are more likely to report symptoms in a negative light, or the association may be a spurious one due to the impact on symptoms and discontinuity of some other variable that was not assessed. All the symptom measures involved the participant’s evaluation of the symptom, and there are concerns about the accuracy of family reports about symptoms (Loewenstein et al., 2001). As noted earlier, the *Communicative Effectiveness Index* did not provide a direct and comprehensive measure of emotional warmth and responsiveness and therefore did not provide a fair test of the hypothesis that this variable may be an important factor in perceptions of continuity/discontinuity. It should also be noted that the *Communicative Effectiveness Index* was designed for use after stroke, and it may not provide the best method of capturing dementia-related changes. Finally, the *Memory* subscale of the *Revised Memory and Behavior Checklist* showed low internal reliability, and the failure to observe a significant correlation between this measure and the *Birmingham Relationship Continuity Measure* should accordingly be treated with some caution.

### Potential implications

Given the potential benefits of continuity in terms of carer well-being and the quality of care they provide (Riley, 2019), it may be useful, in some circumstances at least, to support carers in trying to maintain a sense of continuity. The current study suggests that maintaining continuity may be more difficult in the face of certain symptoms. One potential method of supporting greater continuity involves changing how carers appraise these symptoms. For example appraisals made by carers about challenging interpersonal behaviours such as aggression sometimes involve the perception of the behaviours being under the control of the person with dementia and motivated by hostile intent (Harvath, 1994; Martin-Cook et al., 2003). In the context of a loving pre-dementia relationship, such an interpretation implies a stark contrast between the person and the relationship before and after the onset of dementia and thereby presumably increases the probability of perceiving discontinuity. Such an appraisal may also make it difficult to maintain loving feelings towards the person with dementia. The carer could be supported to develop a more nuanced understanding of the behaviour that does not involve the idea of the person with dementia bearing any sustained personal animosity. Externalising and depersonalising problems are an important component of narrative therapy (White, 2007), and this approach merits further exploration as a way of helping carers avoid personalised interpretations of challenging interpersonal behaviours that may be contributing to a sense of discontinuity.

## References

[bibr1-1471301221994311] Aminzadeh F. Byszewski A. Molnar F. J. Eisner M. (2007). Emotional impact of dementia diagnosis: Exploring persons with dementia and caregivers’ perspectives. Aging & Mental Health, 11, 281-290. 17558579 10.1080/13607860600963695

[bibr2-1471301221994311] Bodley-Scott S. E. M. Riley G. A. (2015). How partners experience personality change after traumatic brain injury – its impact on their emotions and their relationship. Brain Impairment, 16(3), 205-220.

[bibr3-1471301221994311] Boylstein C. Hayes J. (2012). Reconstructing marital closeness while caring for a spouse with Alzheimer’s. Journal of Family Issues, 33(5), 584-612.

[bibr4-1471301221994311] Bucks R. S. Ashworth D. L. Wilcock G. K. Siegfried K. (1996). Assessment of activities of daily living in dementia: Development of the Bristol activities of daily living scale. Age and Ageing, 25(2), 113-120. 8670538 10.1093/ageing/25.2.113

[bibr5-1471301221994311] Chesla C. Martinson I. Muwaswes M. (1994). Continuities and discontinuities in family members’ relationships with Alzheimer’s patients. Family Relations, 43(1), 3-9.

[bibr6-1471301221994311] Cummings J. L. Mega M. Gray K. Rosenberg-Thompson S. Carusi D. A. Gornbein J. (1994). The neuropsychiatric inventory: Comprehensive assessment of psychopathology in dementia. Neurology, 44(12), 2308. 7991117 10.1212/wnl.44.12.2308

[bibr7-1471301221994311] De Vugt M. E. Stevens F. Aalten P. Lousberg R. Jaspers N. Winkens I. Jolles J. Verhey F. R. J. , (2003). Behavioural disturbances in dementia patients and quality of the marital relationship. International Journal of Geriatric Psychiatry, 18(2), 149-154. 12571824 10.1002/gps.807

[bibr8-1471301221994311] Evans D. Lee E. (2014). Impact of dementia on marriage: A qualitative systematic review. Dementia, 13(3), 330-349. 24339060 10.1177/1471301212473882

[bibr9-1471301221994311] Faul F. Erdfelder E. Lang A.-G. Buchner A. (2007). G*Power 3: A flexible statistical power analysis program for the social, behavioral, and biomedical sciences. Behavior Research Methods, 39(2), 175-191. 17695343 10.3758/bf03193146

[bibr10-1471301221994311] Gillies B. (2012). Continuity and loss: The carer’s journey through dementia. Dementia, 11(5), 657-676.

[bibr11-1471301221994311] Harvath T. A. (1994). Interpretation and management of dementia-related behaviour problems. Clinical Nursing Research, 3(1), 7-25. 8167579 10.1177/105477389400300102

[bibr12-1471301221994311] Hendrick S. S. (1988). A generic measure of relationship satisfaction. Journal of Marriage and the Family, 50(1), 93-98.

[bibr13-1471301221994311] Kaplan L. (2001). A couplehood typology for spouses of institutionalized persons with Alzheimer’s disease: Perceptions of ‘We’–‘I’. Family Relations, 50(1), 87-98.

[bibr14-1471301221994311] Lewis R. D. H. (1998). The impact of the marital relationship on the experience of caring for an elderly spouse with dementia. Ageing and Society, 18(2), 209-231.

[bibr15-1471301221994311] Lindauer A. Harvath T. A. (2015). The meanings caregivers ascribe to dementia-related changes in care recipients: A meta-ethnography. Research in Gerontological Nursing, 8(1), 39-48. 25490777 10.3928/19404921-20141121-01

[bibr16-1471301221994311] Loewenstein D.A. Arguelles S. Bravo M. Freeman R.Q. Arguelles T. Acevedo A. Eisdorfer C. (2001). Caregivers’ judgments of the functional abilities of the Alzheimer’s disease patient: A comparison of proxy reports and objective measures. The Journals of Gerontology. Series B, Psychological Sciences and Social Sciences, 56(2), 78-84. 10.1093/geronb/56.2.p78 11245361

[bibr17-1471301221994311] Lomas J. Pickard L. Bester S. Elbard H. Finlayson A. Zoghaib C. (1989). The communicative effectiveness index: Development and psychometric evaluation of a functional communication measure for adult aphasia. The Journal of Speech and Hearing Disorders, 54(1), 113-124. 2464719 10.1044/jshd.5401.113

[bibr18-1471301221994311] Martin-Cook K. Remakel-Davis B. Svetlik D. Hynan L. S. Weiner M. F. (2003). Caregiver attribution and resentment in dementia care. American Journal of Alzheimer’s Disease and Other Dementias, 18(6), 366-374. 10.1177/153331750301800606 PMC10833658 14682086

[bibr19-1471301221994311] McDonald S. Flanagan S. Rollins J. Kinch J. (2003). TASIT: A new clinical tool for assessing social perception after traumatic brain injury. Journal of Head Trauma Rehabilitation, 18(3), 219-238. 10.1097/00001199-200305000-00001 12802165

[bibr20-1471301221994311] Murray J. Livingston G. (1998). A qualitative study of adjustment to caring for an older spouse with psychiatric illness. Ageing and Society, 18(6), 659-671.

[bibr21-1471301221994311] Poveda B. Osborne-Crowley K. Laidlaw K. Macleod F. Power K. (2017). Social cognition, behaviour and relationship continuity in dementia of the Alzheimer type. Brain Impairment, 18(2*),* 175-187.

[bibr22-1471301221994311] Quinn C. Clare L. Woods R. T. (2015). Balancing needs: The role of motivations, meanings and relationship dynamics in the experience of informal caregivers of people with dementia. Dementia, 14(2), 220-237. 24339101 10.1177/1471301213495863

[bibr23-1471301221994311] Riley G. A. (2019). Relationship continuity/discontinuity - a framework for looking at the role of relationships in the experience of living with dementia. American Journal of Alzheimer’s Disease and Other Dementias, 34(3), 145-147. 10.1177/1533317518813557 PMC10852478 30453742

[bibr24-1471301221994311] Riley G. A. Achiampong J. Hillberg T. Oyebode J. R. (2020). Relationship continuity and person-centred care in how spouses make sense of challenging care needs. Aging & Mental Health, 24(2), 242-249. 30415564 10.1080/13607863.2018.1531380

[bibr25-1471301221994311] Riley G. A. Evans L. Oyebode J. R. (2018). Relationship continuity and emotional well-being in spouses of people with dementia. Aging & Mental Health, 22(3), 299-305. 27809565 10.1080/13607863.2016.1248896

[bibr26-1471301221994311] Riley G. A. Fisher G. Hagger B. F. Elliott A. Le Serve H. Oyebode J. R. (2013). The Birmingham relationship continuity measure: The development and evaluation of a measure of the perceived continuity of spousal relationships in dementia. International Psychogeriatrics, 25(2)*,* 263-274. 23174150 10.1017/S1041610212001743

[bibr27-1471301221994311] Spector A. Orrell M. Charlesworth G. Marston L. (2016). Factors influencing the person-carer relationship in people with anxiety and dementia. Aging & Mental Health, 20(10), 1055-1062. 26207801 10.1080/13607863.2015.1063104

[bibr28-1471301221994311] Spruytte N. Audenhove C. Lammertyn F. Storms G. (2002). The quality of the caregiving relationship in informal care for older adults with dementia and chronic psychiatric patients. Psychology and Psychotherapy: Theory, Research and Practice, 75(3)*,* 295-311. 10.1348/147608302320365208 12396755

[bibr29-1471301221994311] Strohminger N. Nichols S. (2014). The essential moral self. Cognition, 131, 159-171. 24503450 10.1016/j.cognition.2013.12.005

[bibr30-1471301221994311] Strohminger N. Nichols S. (2015). Neurodegeneration and identity. Psychological Science, 26(9), 1469-1479. 26270072 10.1177/0956797615592381

[bibr31-1471301221994311] Teri L. Truax P. Logsdon R. Uomoto J. Zarit S. Vitaliano P. P. (1992). Assessment of behavioral problems in dementia: The revised memory and behavior problems checklist. Psychology and Aging, 7(4), 622-631. 1466831 10.1037//0882-7974.7.4.622

[bibr32-1471301221994311] Villa D. Riley G. A. (2017). Partners’ experiences of relationship continuity in acquired brain injury. Cogent Psychology, 4, 1380891.

[bibr33-1471301221994311] Walters A. H. Oyebode J. R. Riley G. A. (2010). The dynamics of continuity and discontinuity for women caring for a spouse with dementia. Dementia, 9(2)*,* 169-189.

[bibr34-1471301221994311] White M. (2007). Maps of narrative practice. New York: Norton.

